# Trend in Alcohol-related Crashes Before and After the Introduction of Mandatory Breath Testing Among Commercial Truck Drivers

**DOI:** 10.2188/jea.JE20220054

**Published:** 2023-11-05

**Authors:** Masao Ichikawa, Haruhiko Inada, Kota Katanoda, Shinji Nakahara

**Affiliations:** 1 Department of Global Public Health, Faculty of Medicine, University of Tsukuba, Tsukuba, Ibaraki, Japan; 2 Johns Hopkins International Injury Research Unit, Department of International Health, Johns Hopkins Bloomberg School of Public Health, Baltimore, Maryland, USA; 3 Division of Surveillance and Policy Evaluation, National Cancer Center Institute for Cancer Control, Tokyo, Japan; 4 Graduate School of Health Innovation, Kanagawa University of Human Services, Kawasaki, Kanagawa, Japan

**Keywords:** commercial driver, drunk driving, motor vehicle collision, prevention

## Abstract

**Background:**

Since 2011, commercial truck drivers have been required to take alcohol breath tests at the beginning and end of their working hours due to their employers’ legal obligations. However, non-commercial truck drivers are not required to do so. We examined whether alcohol-related crashes had decreased after 2011 among commercial truck drivers.

**Methods:**

Using police data, we conducted a joinpoint regression analysis to examine the trend in the proportion of alcohol-related crashes from 1995 through 2020 caused by commercial truck drivers (who were subjected to alcohol breath testing) and non-commercial truck drivers (who were not subjected to testing). The annual percentage change in this trend was also estimated.

**Results:**

During the 26-year study period, truck drivers caused 1,846,321 at-fault crashes, and 0.4% of the crashes involved intoxicated driving. A significant decreasing trend in the proportion of alcohol-related crashes was identified among both commercial and non-commercial truck drivers in the 2000s, during which several legal amendments were made against drunk driving. The annual percentage change was −13.5% from 2001 to 2012 among commercial truck drivers, and −14.9% from 2001 to 2011 among non-commercial truck drivers. No decreasing trend was observed afterwards, despite the introduction of mandatory alcohol breath testing in 2011.

**Conclusion:**

The effect of mandatory alcohol breath testing on reducing alcohol-related crashes among commercial truck drivers was not evident.

## INTRODUCTION

Driving under the influence of alcohol increases the risk of motor vehicle crashes, but drunk driving is persistent globally. According to the Global Status Report on Road Safety 2018, the proportion of road traffic deaths attributable to alcohol was 5.6% in Japan, and 61 of 94 countries that had the data on it reported the proportion >5.6% (largely between 10% and 40%).^1^ To prevent alcohol-related crashes, a growing number of countries have adopted and increasingly enforced legislative restrictions on drunk driving.^1^

In Japan, the law against drunk driving was especially tightened in the 2000s, following high-profile crashes caused by intoxicated drivers, which subsequently induced extensive public debates demanding stricter rules and penalties for drunk driving.^2^^,^^3^ Consequently, the Road Traffic Act was amended in 2002 and 2007 to lower the punishable limit of blood alcohol concentration from 0.5 mg/mL to 0.3 mg/mL, with a large increase in fines and penalty points, and prolonged periods of license suspension and revocation. The Penal Code (criminal law) was also amended in 2001 and 2004 to punish dangerous driving causing death or injury using imprisonment or fines. These legal amendments and improved social norms against drunk driving led to a reduction in alcohol-related crashes in the 2000s.^3^^,^^4^

To prevent drunk driving, an additional countermeasure was taken in the commercial transportation sector.^5^ Since May 2011, the employers of commercial drivers who carry passengers for a fee (ie, bus and taxi drivers) or goods for their customers (ie, truck drivers, hereinafter referred to as commercial truck drivers), have been obligated by law to check employees’ sobriety through alcohol breath tests at the beginning and end of their shifts. In cases where drivers begin or end their work away from the office, they are required to carry portable breathalyzers and report to their affiliated offices by phone that they are sober, or they may be checked at the branch of their affiliated offices.

The evaluation of the effect of mandatory breath testing on alcohol-related crashes is indispensable to leverage testing as an effective countermeasure against drunk driving among commercial drivers. With the small number of alcohol-related crashes among bus and taxi drivers, quantitative evaluation is only practical for commercial truck drivers. Notably, the employers of truck drivers who carry their companies’ goods—that is, of non-commercial truck drivers—are not obligated to check employees’ sobriety using alcohol breath tests; thus, non-commercial truck drivers can be a comparison group in the evaluation. To date, the effect of alcohol breath testing has not been evaluated among commercial truck drivers or other commercial drivers. Nevertheless, the government decided to extend the testing to the employers of non-commercial drivers—if they operate five or more vehicles, or one vehicle with a seating capacity of 11 or more people—from October 2022, following a high-profile crash caused by an intoxicated non-commercial truck driver in June 2021.^6^ In this crash, the driver drove into five children on their way to school, two of whom were killed. To prevent such tragic events, the effectiveness of countermeasures against drunk driving should be verified, and the effect of mandatory alcohol breath testing should be evaluated before its extension. The present study, therefore, examines the trend of alcohol-related crashes among truck drivers before and after the introduction of breath testing for commercial truck drivers.

## METHODS

### Data

We obtained at-fault crash data among truck drivers—spanning 1995–2020—from the Institute for Traffic Accident Research and Data Analysis, which compiled traffic crash data from the National Policy Agency. Data from 1995 were obtained because information on the occupational classification of commercial and non-commercial drivers was only available from 1995. The data included the drivers’ alcohol intoxication during the crash, occupation (commercial or non-commercial drivers), designation of operation managers in their companies, size and designated use of their truck in the crash (commercial or non-commercial use), and the highest severity of injury caused by the crash (fatal, serious, or minor). A fatal injury is defined as a death that occurred within 24 hours from the crash, while a serious injury is an injury requiring treatment for at least 30 days as estimated by the physician; all other instances are considered a minor injury.

Drivers with any level of alcohol intoxication were considered to have committed drunk driving because driving with any level of alcohol intoxication is prohibited in Japan.^7^ Drivers will be penalized if the breath test indicates blood alcohol concentration ≥0.3 mg/mL, or if police investigation determines that drivers are under the influence of alcohol regardless of the alcohol concentration. Driving with blood alcohol concentration <0.3 mg/mL is still illegal, though not punished based on the breath test result alone. Operation managers are assigned to supervise drivers’ safety and schedules; it is a regulatory requirement to designate operation managers for any commercial driver company and for companies with non-commercial drivers—if they operate five or more vehicles, or one vehicle with a seating capacity of 11 or more people.

Trucks of various sizes are designed to carry goods in their cargo areas. Since the size definition changed twice during the study period, three categories were created to be consistent throughout the study period: large (vehicle weight of 11 tons or above, or loading capacity of 6.5 tons or above), medium/ordinary (other than large or light vehicles), and light (engine replacement up to 660 cc and loading capacity of 350 kg or below). Vehicles designated for commercial use must be used for commercial transportation, and only commercial drivers must drive. Commercial drivers may drive non-commercial vehicles if they carry their companies’ goods exclusively (ie, for non-commercial transportation). Commercial and non-commercial vehicles have different colored registration plates; therefore, the designated use of vehicles is recognizable at a glance.

In addition to the crash data of truck drivers, we obtained the number of total crashes and alcohol-related crashes caused by all vehicle drivers (including four-wheeled vehicles, motorcycles, and moped drivers) in each year of the study period from the National Policy Agency.^8^ This study using aggregate public domain data did not require an institutional review board approval.

### Analysis

We analyzed annual crash data because the number of alcohol-related crashes was too small to analyze on a monthly basis. The average monthly number of alcohol-related crashes caused by commercial and non-commercial truck drivers during the 26-year period was 8 and 13, respectively, and this number is much smaller in recent years of the study period. We considered the study period from 2011 onwards as the post-intervention period because mandatory alcohol breath testing was introduced in May 2011, and companies might have started testing prior to the month of the introduction. The number of crashes caused by commercial truck drivers while driving non-commercial vehicles was added to the number of crashes caused by non-commercial truck drivers because commercial truck drivers were not required to take alcohol breath tests by law when they drove non-commercial vehicles. Moreover, the number of alcohol-related crashes caused by commercial truck drivers while driving non-commercial vehicles was too small to compare with those caused by commercial truck drivers while driving commercial vehicles. For simplicity, commercial truck drivers who caused crashes when driving non-commercial vehicles are categorized as non-commercial truck drivers. Then, we described the characteristics of crashes caused by commercial and non-commercial truck drivers separately, regarding the size of their trucks, the designation of operation managers in their companies, the highest severity of injury caused by their crashes, and whether they were intoxicated during the crashes.

To examine the trend of alcohol-related crashes, we calculated—separately for each year of the study period—the proportion of alcohol-related crashes to all crashes caused by commercial and non-commercial truck drivers. The proportion of alcohol-related crashes was also calculated for the entire population of vehicle drivers. Crashes in which driver alcohol intoxication was not determined were excluded from the denominator of this proportion. Since such crashes accounted for only 0.09% of total crashes, the results would not be distorted by excluding them from the analysis.

We conducted a joinpoint regression analysis to examine the trend in the proportion of alcohol-related crashes over the 26-year study period using Joinpoint version 4.9.0.0 (Statistical Methodology and Applications Branch, Surveillance Research Program, National Cancer Institute, Bethesda, MD, USA).^9^^,^^10^ The proportion of alcohol-related crashes to all crashes was used as an outcome variable rather than the rate of alcohol-related crashes per kilometer driven or per driver population because such information was not readily available for truck drivers. Therefore, we assumed that the total number of crashes represented the total distance driven or the amount of risk exposures.

In the joinpoint regression analysis, potential change-points in the trend are not predefined, but the best-fit lines are identified, and the points where the line segments are joined (called joinpoints) indicate change-points in the trend. The analysis starts with testing no joinpoints in the trend, then adding joinpoints based on a permutation test to identify the best-fit lines. In the present study, a maximum of four joinpoints were allowed, given 26 data points. Using a log-linear model with the proportion of alcohol-related crashes as the dependent variable and its standard error in each year of the study period, the joinpoint regression analysis determined the year of trend change, the annual percent change in each period of the trend (divided by the year of trend change), and its 95% confidence interval (CI). The annual percent change is significantly different from zero at the alpha level of 0.05 if its 95% CI does not include zero, which is hereinafter referred to as a significant trend. If mandatory breath testing was effective in reducing alcohol-related crashes, a trend change should be observed after 2011—that is, when testing was introduced. Using the joinpoint regression analysis, we considered the impact of legal amendments against drunk driving during the 2000s in addition to testing. The analysis was repeated separately for fatal and serious injury crashes and minor injury crashes among truck drivers to examine a potential difference in the trend of alcohol-related crashes by the crash severity.

Additionally, we conducted a sensitivity analysis among truck drivers, using the number of alcohol-related crashes as an outcome variable in the joinpoint regression analysis. In this analysis, Poisson variance was assumed. Furthermore, to consider the secular trend of alcohol-related crashes among truck drivers (ie, factors influencing alcohol-related crashes and total crashes that might have changed over the study period, such as alcohol consumption, automotive safety, and traffic volume and environment), we calculated the ratio of the proportion of alcohol-related crashes caused by commercial truck drivers (who were subjected to alcohol breath testing) to those caused by non-commercial truck drivers (who were not subjected to testing). We then conducted a joinpoint regression analysis to examine the trend in this ratio. In this analysis, constant variance was assumed. If mandatory alcohol breath testing is effective, the ratio should abruptly decrease; thus, the trend should change after 2011—that is, when testing was introduced.

## RESULTS

In the 26-year study period, there were a total of 1,846,321 at-fault crashes among truck drivers, including 662,396 crashes caused by commercial truck drivers, and 1,183,925 crashes caused by non-commercial truck drivers (of which, 90,096 crashes were caused by commercial truck drivers while driving non-commercial vehicles). Table 1 shows the characteristics of crashes caused by commercial and non-commercial truck drivers. The size of their trucks was mainly medium/ordinary among both types of truck drivers (61% and 67%, respectively), but the proportion of large vehicles was much greater among commercial truck drivers (27%) than non-commercial truck drivers (3%). Operation managers were designated in 78% of the companies with commercial truck drivers, and 32% of the companies with non-commercial truck drivers, while 60% of the companies with non-commercial truck drivers were not required to designate operation managers due to the small number of vehicles they operated. The proportion of fatal crashes to all crashes caused by commercial and non-commercial truck drivers was 1.9% and 1.0%, respectively. The proportion of alcohol-related crashes to all crashes caused by commercial and non-commercial truck drivers was 0.4% and 0.3%, respectively, whereas it was 1.7% among all vehicle drivers (data not shown).

**Table 1.** Characteristics of at-fault crashes caused by commercial and non-commercial truck drivers from 1995–2020

	Commercial truck drivers		Non-commercial truck drivers	
	
	*n*	%	*n*	%
Total number of crashes	662,396		1,183,925	

Drivers’ truck size^a^				
Large	176,550	26.7%	38,650	3.3%
Medium/Ordinary	404,675	61.1%	798,199	67.4%
Light	81,171	12.3%	347,076	29.3%

Operation mangers in drivers’ companies^b^				
Designated	516,266	77.9%	378,280	32.0%
Not designated despite the requirement	130,003	19.6%	76,538	6.5%
Not required	n/a		709,106	59.9%
Undetermined	16,127	2.4%	20,001	1.7%

Highest severity of injury in the crash				
Fatal	12,660	1.9%	11,765	1.0%
Serious	50,409	7.6%	83,978	7.1%
Minor	599,327	90.5%	1,088,182	91.9%

Drivers’ alcohol intoxication				
Intoxicated	2,577	0.4%	4,071	0.3%
Non-intoxicated	659,276	99.5%	1,178,830	99.6%
Undetermined	543	0.1%	1024	0.1%

^a^“Large” truck size is defined as having a vehicle weight of 11 tons or above, or loading capacity of 6.5 tons or above; “medium/ordinary” is defined as those other than large or light vehicles, and “light” is defined as having an engine replacement up to 660 cc and a loading capacity of 350 kg or below.

^b^The designation of operation managers to supervise drivers’ safety and schedules is required for all companies of commercial drivers and for companies of non-commercial drivers—if they operate five or more vehicles, or one vehicle with a seating capacity of 11 or more people.

**Figure 1.**
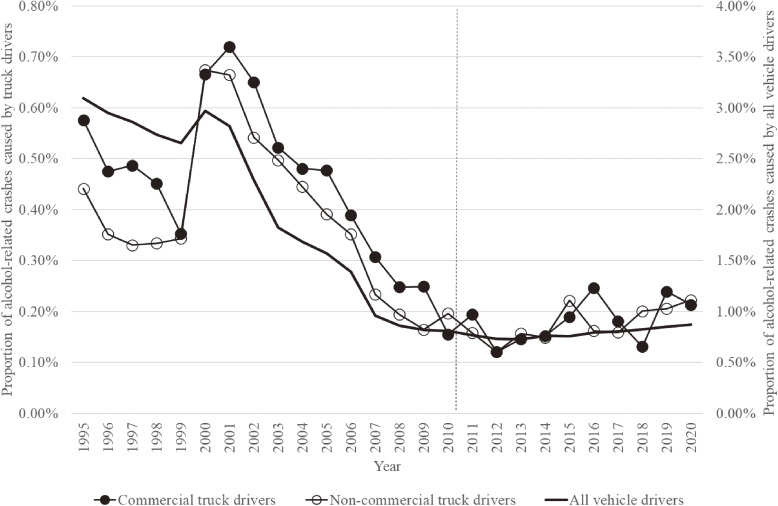
Proportion of alcohol-related crashes caused by commercial and non-commercial truck drivers and all vehicle drivers from 1995–2020. During the 2000s, several legal amendments were made against drunk driving. Since May 2011, commercial truck drivers have been required to take alcohol breath tests at the beginning and end of their working hours. All vehicle drivers include four-wheeled vehicles, motorcycles, and moped drivers.

Figure 1 shows the overall trend in the proportion of alcohol-related crashes caused by commercial and non-commercial truck drivers and all vehicle drivers during the study period. This proportion, which abruptly increased from 1999 to 2000 by approximately 0.3% point among these three groups of drivers, decreased substantially throughout the 2000s. However, this decreasing trend stagnated during the 2010s—not only among commercial truck drivers, but also among non-commercial truck drivers and all vehicle drivers. The number of at-fault crashes by driver alcohol intoxication and the proportion of alcohol-related crashes caused by the three groups of drivers in each year of the study period is shown in eTable 1.

Table 2 shows the annual percentage change and its 95% CI in each period of the trend in the proportion of alcohol-related crashes from 1995 to 2020. Among commercial truck drivers, a trend change was observed in 1998, 2001, and 2012. The proportion showed a decreasing trend of −10.6% (95% CI, −21.7 to 2.0%) until 1998, followed by an increasing trend of 22.6% (95% CI, −6.4 to 60.5%) until 2001, then a significant decreasing trend of −13.5% (95% CI, −15.8 to −11.1%) until 2012, and an increasing trend of 5.1% (95% CI, −1.7 to 12.3%) until 2020. Similarly, among non-commercial truck drivers, a trend change was observed in 1998, 2001, and 2011. The proportion showed a decreasing trend of −8.5% (95% CI, −19.0 to 3.4%) until 1998, followed by a significant increasing trend of 30.2% (95% CI, 4.1 to 62.8%) until 2001, then a significant decreasing trend of −14.9% (95% CI, −17.3 to −12.4%) until 2011, and an increasing trend of 4.9% (95% CI, −0.2 to 10.4%) until 2020. Among all vehicle drivers, a trend change was observed in 2001 and 2009. The proportion showed a decreasing trend of −1.4% (95% CI, −3.5 to 0.7%) until 2001, then to a steeper significant decreasing trend of −14.6% (95% CI, −16.7 to −12.5%) until 2009, and followed by an increasing trend of 0.5% (95% CI, −1.6 to 2.7%) until 2020. The observed and predicted trends among the three groups of drivers are shown in eFigure 1.

**Table 2.** Annual percent change and its 95% confidence interval in each period of the trend in the proportion of alcohol-related crashes caused by commercial and non-commercial truck drivers and all vehicle drivers, from 1995–2020^a^

Commercial truck drivers			Non-commercial truck drivers			All vehicle drivers^b^		
		
Period^c^	APC	95% CI	Period^c^	APC	95% CI	Period^c^	APC	95% CI
1995–1998	−10.6	−21.7 to 2.0	1995–1998	−8.5	−19.0 to 3.4	1995–2001	−1.4	−3.5 to 0.7
1998–2001	22.6	−6.4 to 60.5	1998–2001^d^	30.2	4.1 to 62.8			
2001–2012^d^	−13.5	−15.8 to −11.1	2001–2011^d^	−14.9	−17.3 to −12.4	2001–2009^d^	−14.6	−16.7 to −12.5
2012–2020	5.1	−1.7 to 12.3	2011–2020	4.9	−0.2 to 10.4	2009–2020	0.5	−1.6 to 2.7

APC, annual percent change; CI, confidence interval.

^a^Since May 2011, commercial truck drivers have been required to take alcohol breath tests at the beginning and end of their working hours.

^b^Including four-wheeled vehicles, motorcycles, and moped drivers.

^c^Divided by the year of trend change identified in joinpoint regression analysis.

^d^The APC in this period is significantly different from zero at the alpha level of 0.05.

The abovementioned results are shown in eTable 2 separately for fatal and serious injury crashes and minor injury crashes among truck drivers. In the proportion of alcohol-related fatal and serious injury crashes, a trend change was observed in 1997, 2003, and 2010 among commercial truck drivers, with a significant increasing and decreasing trend from 1997 to 2003 and from 2003 to 2010, respectively, whereas a significant decreasing trend over the entire study period (thus, no trend change) was observed among non-commercial truck drivers. In the proportion of alcohol-related minor injury crashes, there was a trend change in 2002 and 2012 among commercial truck drivers with a significant decreasing trend from 2002 to 2012, and a trend change in 1997, 2001, and 2011 among non-commercial truck drivers with a significant increasing and decreasing trend from 1997 to 2001 and from 2001 to 2011, respectively. The observed and predicted trends are shown in eFigure 2.

In the sensitivity analysis using the number of alcohol-related crashes among truck drivers, the year of trend change and significant and non-significant trends in each period of the trend remained the same among both commercial and non-commercial truck drivers (eTable 3). In the analysis that considered the secular trend of alcohol-related crashes among truck drivers, using the ratio of the proportion of alcohol-related crashes caused by commercial truck drivers to those caused by non-commercial truck drivers, we observed a significant decreasing trend over the entire study period with the annual percent change of −1.1% (95% CI, −2.1 to −0.1%), in which no trend change was identified (eFigure 3).

## DISCUSSION

Alcohol-related crashes among commercial truck drivers did not decrease after 2011 when mandatory breath testing was introduced to them, irrespective of the crash severity. During the study period (1995–2020), the proportion of alcohol-related crashes to all crashes caused by commercial truck drivers started to decrease in 2001, constantly decreasing until 2012, but stagnated almost concurrently with the introduction of mandatory breath testing in 2011. This finding was supported by the sensitivity analysis using the number of alcohol-related crashes. Moreover, the similar trend was observed in the crashes caused by non-commercial truck drivers and all vehicle drivers. It is, therefore, unlikely that mandatory breath testing would help prevent drunk driving among commercial truck drivers in its current form of administration; commercial truck drivers’ sobriety is only checked at the beginning and end of working hours at the office, or self-reported when they begin or end their work away from the office.

The decreasing trend of alcohol-related crashes stagnated in the 2010s, possibly because adaptable drivers had already begun refraining from drunk driving in the 2000s, during which stringent legal amendments were made against drunk driving, although a few intractable drivers continued drunk driving in the 2010s. Such intractable drivers might not have responded to mandatory breath testing only at the beginning and end of their working hours at the office, or through their self-reports away from the office. Apparently, drunk driving is still possible during working hours, especially when they begin or end their work day away from the office.

Prior to the decreasing trend in the 2000s, there was an abrupt increase from 1999 to 2000 in the proportion of alcohol-related crashes among both truck drivers and all vehicle drivers to almost the same extent (by approximately 0.3% point). However, the joinpoint regression analysis identified a trend change with this increase only among truck drivers possibly because of the different level of changes (from 0.35% to 0.67% and from 0.34% to 0.67% among commercial and non-commercial truck drivers, respectively, and from 2.65% to 2.97% among all vehicle drivers). The abrupt increase may be attributable to an improved police investigation into driver alcohol intoxication following successive high-profile crashes caused by an intoxicated truck driver in November 1999 and by an intoxicated unlicensed driver in April 2000.^11^

From the perspective of evidence-based traffic policies, the effectiveness of mandatory breath testing in the current or any other form of administration should be formally evaluated if testing is continued. Otherwise, alternative countermeasures that have proven to be effective must be considered. A potential countermeasure is the installation of an ignition interlock device. To start a vehicle with this device, drivers need to breathe into the device, and if the alcohol content is beyond the limit, the vehicle will not start. A systematic review of evidence from several countries suggests that the device is effective as a method of preventing drunk driving among previous offenders.^12^ A recent nationwide study in the United States reported that state laws requiring the device installation for offenders were associated with a 25% reduction in alcohol-related fatal crashes.^13^ In Japan, the Ministry of Land, Infrastructure and Transport developed and released technical guidelines on an ignition interlock device in 2012,^14^ and the device is available in the market. However, the device has not been used as a legislative countermeasure against drunk driving. Therefore, testing the device in Japan among truck drivers who might have alcohol-related problems is a worthwhile endeavor.

Alcohol-related crashes tend to be more fatal, often killing those involved in the collision^15^; therefore, there is a social need for efforts to eliminate such crashes. In late 1999 and early 2000, when high-profile alcohol-related crashes occurred, extensive public debates arose, and a petition calling for stricter rules and penalties against drunk driving collected over 370,000 signatures, which led to the subsequent legal amendments.^3^ It is important to note that the countermeasures adopted then were proven to be effective, such as lowered blood alcohol concentration, increased fines, and prolonged license suspension and imprisonment.^16^ Therefore, these legal amendments, combined with public concern, appeared to be effective in reducing alcohol-related crashes. On the other hand, the government’s recent decision to extend mandatory alcohol breath testing to non-commercial drivers as well as the current testing among commercial drivers were neither data-driven nor evidence-based. Given our negative findings, this decision should be reconsidered.

The strength of this study is the use of national data, which made it possible to examine the countermeasures against a relatively small number of events (ie, alcohol-related crashes caused by truck drivers). However, a limitation of this study is the lack of information for further analysis. To prevent commercial drivers from drunk driving, it is essential to examine the characteristics of drivers causing alcohol-related crashes and their companies. The identification of high-risk groups of drivers and companies for drunk driving should be useful for the countermeasure to be effective and efficient, given the small number of alcohol-related crashes relative to the total number of crashes.

In conclusion, the effect of mandatory alcohol breath testing was not evident in reducing alcohol-related crashes among commercial truck drivers. Alternative countermeasures against drunk driving should be considered to prevent alcohol-related crashes.

## SUPPLEMENTARY MATERIAL

eTable 1. Number of at-fault crashes by driver alcohol intoxication and the proportion of alcohol-related crashes caused by commercial and non-commercial truck drivers and all vehicle drivers, from 1995–2020^a^

eTable 2. APC and its 95% CI in each period of the trend in the proportion of alcohol-related fatal and serious injury crashes and alcohol-related minor injury crashes caused by commercial and non-commercial truck drivers, from 1995–2020^a^

eTable 3. APC and its 95% CI in each period of the trend in the number of alcohol-related crashes caused by commercial and non-commercial truck drivers, from 1995–2020^a^

eFigure 1. Observed and predicted trend (dots and solid line) in the proportion of alcohol-related crashes caused by (**A**) commercial truck drivers, (**B**) non-commercial truck drivers, and (**C**) all vehicle drivers from 1995–2020. During the 2000s, several legal amendments were made against drunk driving. Since May 2011, commercial truck drivers have been required to take alcohol breath tests at the beginning and end of their working hours. All vehicle drivers include four-wheeled vehicles, motorcycles, and moped drivers.

eFigure 2. Observed and predicted trend (dots and solid line) in the proportion of alcohol-related fatal or serious injury crashes caused by (**A**) commercial truck drivers and (**B**) non-commercial truck drivers, and that of alcohol-related minor injury crashes caused by (**C**) commercial truck drivers and (**D**) non-commercial truck drivers from 1995–2020. During the 2000s, several legal amendments were made against drunk driving. Since May 2011, commercial truck drivers have been required to take alcohol breath tests at the beginning and end of their working hours.

eFigure 3. Observed and predicted trend (dots and solid line) in the ratio of the proportion of alcohol-related crashes caused by commercial truck drivers to those caused by non-commercial truck drivers from 1995–2020. During the 2000s, several legal amendments were made against drunk driving. Since May 2011, commercial truck drivers have been required to take alcohol breath tests at the beginning and end of their working hours. All vehicle drivers include four-wheeled vehicles, motorcycles, and moped drivers.
