# Supplementary material for: Trend in Alcohol-related Crashes Before and After the Introduction of Mandatory Breath Testing Among Commercial Truck Drivers
Source: J Epidemiol. 2023 Nov 5;33(11):556–61. doi: 10.2188/jea.JE20220054 (PMC10518377; doi:10.2188/jea.JE20220054)
Supplement: Supplementary file 1 [file je-33-556-s001.pdf]

eTable 1. Number of at-fault crashes by driver alcohol intoxication and the proportion of alcohol-related crashes caused by commercial and non-commercial truck drivers and all vehicle drivers, from 1995–2020[a]

	Commercial truck drivers				Non-commercial truck drivers				All vehicle drivers[b]			
	Intoxicated	Non-intoxicated	Undetermined	%[c]	Intoxicated	Non-intoxicated	Undetermined	%[c]	Intoxicated	Non-intoxicated	Undetermined	%[c]
1995	167	28,817	29	0.58%	277	62,472	90	0.44%	22,346	699,602	1,739	3.10%
1996	139	29,113	35	0.48%	216	61,225	94	0.35%	21,628	711,671	1,889	2.95%
1997	145	29,637	30	0.49%	200	60,317	69	0.33%	21,289	723,257	1,761	2.86%
1998	132	29,137	36	0.45%	196	58,495	101	0.33%	21,060	747,916	2,050	2.74%
1999	110	31,074	40	0.35%	208	60,401	84	0.34%	21,602	792,215	1,995	2.65%
2000	230	34,298	45	0.67%	430	63,373	97	0.67%	26,280	859,414	2,430	2.97%
2001	251	34,643	55	0.72%	413	61,744	95	0.66%	25,400	875,487	2,226	2.82%
2002	220	33,583	28	0.65%	322	59,095	50	0.54%	20,328	868,035	1,690	2.29%
2003	180	34,287	23	0.52%	294	58,823	44	0.50%	16,374	882,102	1,485	1.82%
2004	166	34,373	22	0.48%	263	58,871	27	0.44%	15,178	884,666	1,275	1.69%
2005	162	33,810	31	0.48%	223	56,780	42	0.39%	13,875	868,431	1,258	1.57%
2006	125	32,003	14	0.39%	190	53,850	36	0.35%	11,627	826,490	975	1.39%
2007	75	24,348	7	0.31%	108	46,095	29	0.23%	7,562	778,888	781	0.96%
2008	66	26,528	15	0.25%	87	44,689	20	0.19%	6,219	716,794	600	0.86%
2009	57	22,822	7	0.25%	67	40,621	18	0.16%	5,726	691,859	544	0.82%
2010	36	23,162	12	0.16%	78	39,634	18	0.20%	5,561	681,835	515	0.81%
2011	44	22,598	11	0.19%	60	37,966	16	0.16%	5,030	650,477	460	0.77%
2012	26	21,553	18	0.12%	45	37,231	11	0.12%	4,605	625,648	472	0.73%
2013	30	20,508	10	0.15%	55	35,003	14	0.16%	4,334	591,915	409	0.73%
2014	30	19,659	8	0.15%	48	32,117	11	0.15%	4,155	539,753	371	0.76%
2015	34	17,915	8	0.19%	66	29,731	10	0.22%	3,864	505,882	304	0.76%
2016	41	16,576	11	0.25%	45	27,739	5	0.16%	3,757	470,715	304	0.79%
2017	30	16,520	9	0.18%	43	26,993	10	0.16%	3,582	443,197	310	0.80%
2018	21	15,980	16	0.13%	50	24,839	13	0.20%	3,355	403,129	271	0.83%
2019	34	14,169	9	0.24%	46	22,336	11	0.21%	3,047	354,483	291	0.85%
2020	26	12,163	14	0.21%	41	18,390	9	0.22%	2,522	286,216	257	0.87%

[a]Since May 2011, commercial truck drivers have been required to take alcohol breath tests at the beginning and end of their working hours.

[b]Including four-wheeled vehicles, motorcycles, and moped drivers.

[c]Proportion of alcohol-related crashes. Crashes in which driver alcohol intoxication was not determined were excluded from the denominator of this proportion.

eTable 2. APC and its 95% CI in each period of the trend in the proportion of alcohol-related fatal and serious injury crashes and alcohol-related minor injury crashes caused by commercial and non-commercial truck drivers, from 1995–2020[a]

Fatal and serious injury crashes						Minor injury crashes					
Commercial truck drivers			Non-commercial truck drivers			Commercial truck drivers			Non-commercial truck drivers		
Period[b]	APC	95% CI	Period[b]	APC	95% CI	Period[b]	APC	95% CI	Period[b]	APC	95% CI
1995–1997	–23.1	–43.7–5.0	1995–2020[c]	–2.7	–4.1 to –1.3	1995–2002	5.9	–0.1 to 12.3	1995–1997	–21.1	–43.9 to 11.0
1997–2003[c]	9.1	1.4–17.4							1997–2001[c]	28.4	9.9–50.1
2003–2010[c]	–14.0	–20.1 to –7.5				2002–2012[c]	–14.7	–19.7 to –9.3	2001–2011[c]	–15.5	–18.8 to –12.1
2010–2020	3.7	–1.4 to 9.1				2012–2020	5	–7.5 to 19.2	2011–2020	4.4	–2.8 to 12.2

APC, annual percent change; CI, confidence interval.

[a]Since May 2011, commercial truck drivers have been required to take alcohol breath tests at the beginning and end of their working hours.

[b]Divided by the year of trend change identified in joinpoint regression analysis.

[c]The APC in this period is significantly different from zero at the alpha level of 0.05.

eTable 3. APC and its 95% CI in each period of the trend in the number of alcohol-related crashes caused by commercial and non-commercial truck drivers, from 1995–2020[a]

Commercial truck drivers			Non-commercial truck drivers		
Period[b]	APC	95% CI	Period[b]	APC	95% CI
1995–1998	–10.3	–23.9 to 5.7	1995–1998	–10.3	–23.2 to 4.9
1998–2001	32.1	–5.5 to 84.7	1998–2001[c]	34.0	0.8–78.1
2001–2012[c]	–17.4	–20.1 to –14.5	2001–2011[c]	–18.8	–21.7 to –15.8
2012–2020	–1.8	–9.6 to 6.7	2011–2020	–2.6	–8.7 to 3.8

APC, annual percent change; CI, confidence interval.

[a]Since May 2011, commercial truck drivers have been required to take alcohol breath tests at the beginning and end of their working hours.

[b]Divided by the year of trend change identified in joinpoint regression analysis.

[c]The APC in this period is significantly different from zero at the alpha level of 0.05.

(A)

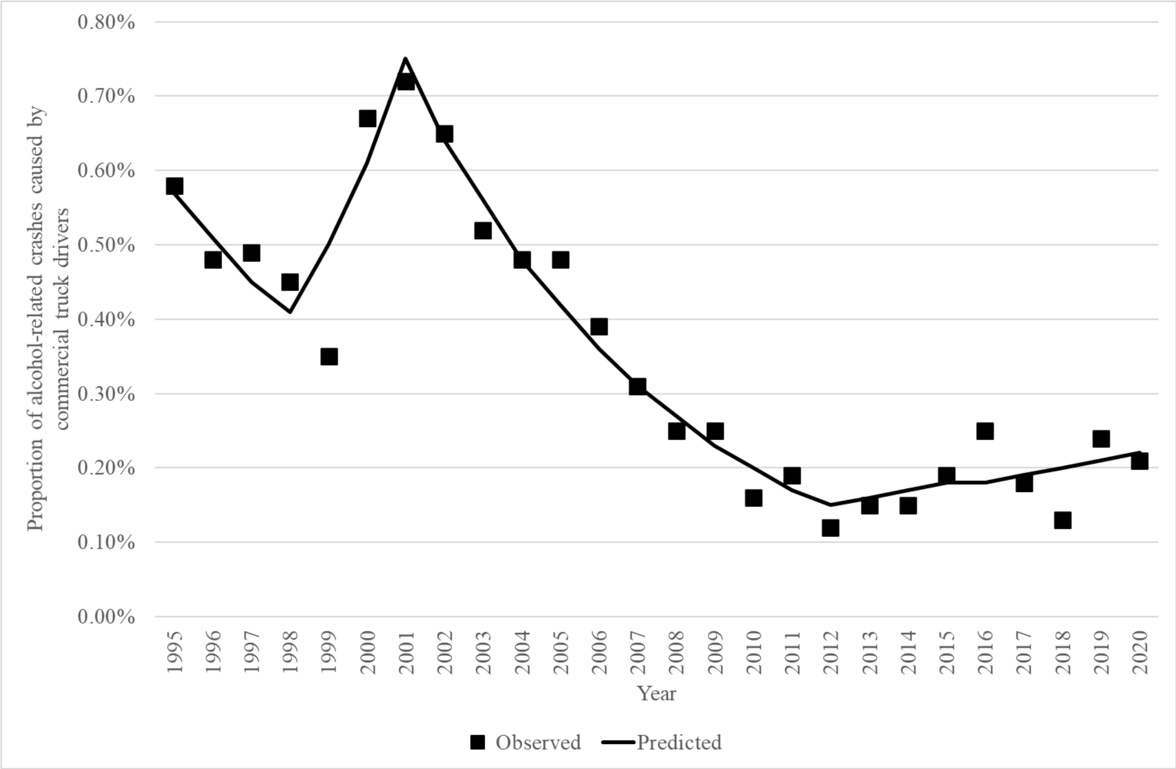
0.80%
0.70%
0.60%
0.50%
0.40%
0.30%
0.20%
0.10%
0.00%
Proportion of alcohol-related crashes caused by commercial truck drivers
1995 1996 1997 1998 1999 2000 2001 2002 2003 2004 2005 2006 2007 2008 2009 2010 2011 2012 2013 2014 2015 2016 2017 2018 2019 2020
Year
Observed
Predicted

(B)

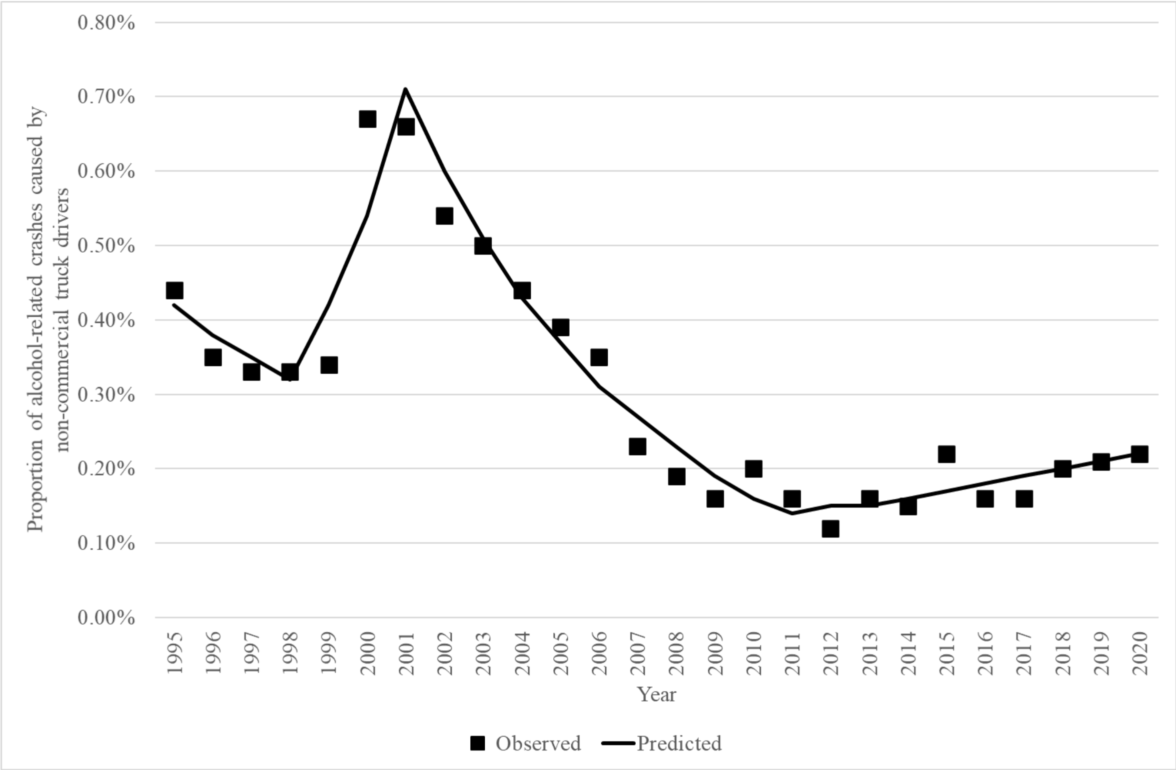
0.80%
0.70%
0.60%
0.50%
0.40%
0.30%
0.20%
0.10%
0.00%
Proportion of alcohol-related crashes caused by non-commercial truck drivers
1995 1996 1997 1998 1999 2000 2001 2002 2003 2004 2005 2006 2007 2008 2009 2010 2011 2012 2013 2014 2015 2016 2017 2018 2019 2020
Year
Observed
Predicted

(C)

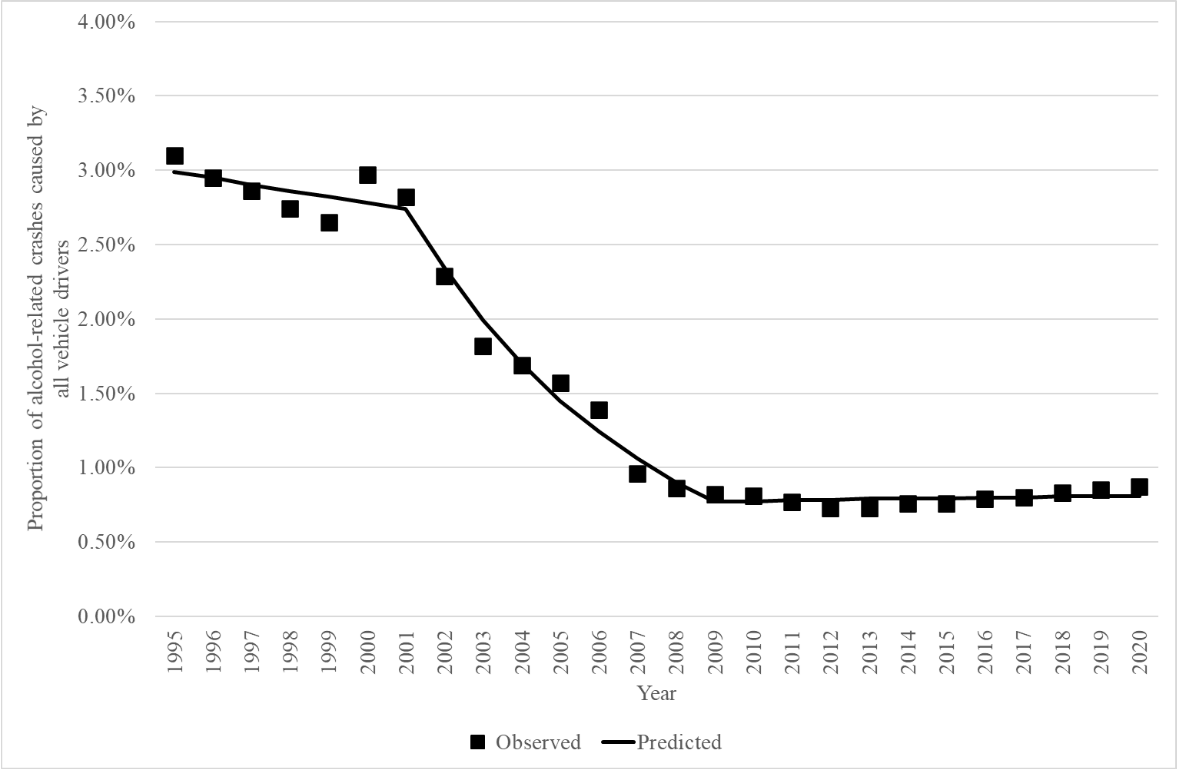

eFigure 1. Observed and predicted trend (dots and solid line) in the proportion of alcohol-related crashes caused by (**A**) commercial truck drivers, (**B**) non-commercial truck drivers, and (**C**) all vehicle drivers from 1995–2020. During the 2000s, several legal amendments were made against drunk driving. Since May 2011, commercial truck drivers have been required to take alcohol breath tests at the beginning and end of their working hours. All vehicle drivers include four-wheeled vehicles, motorcycles, and moped drivers.

(A)

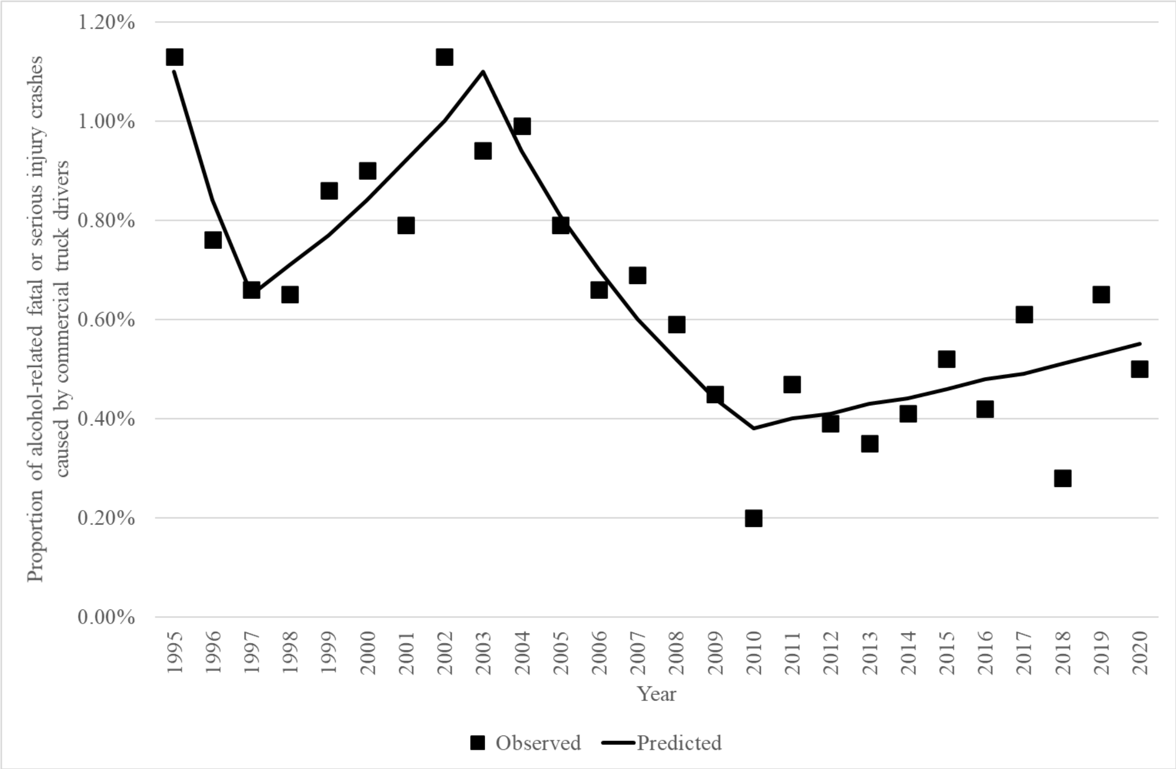

1.20%
1.00%
0.80%
0.60%
0.40%
0.20%
0.00%
Proportion of alcohol-related fatal or serious injury crashes caused by commercial truck drivers
1995
1996
1997
1998
1999
2000
2001
2002
2003
2004
2005
2006
2007
2008
2009
2010
2011
2012
2013
2014
2015
2016
2017
2018
2019
2020
Year
Observed
Predicted

(B)

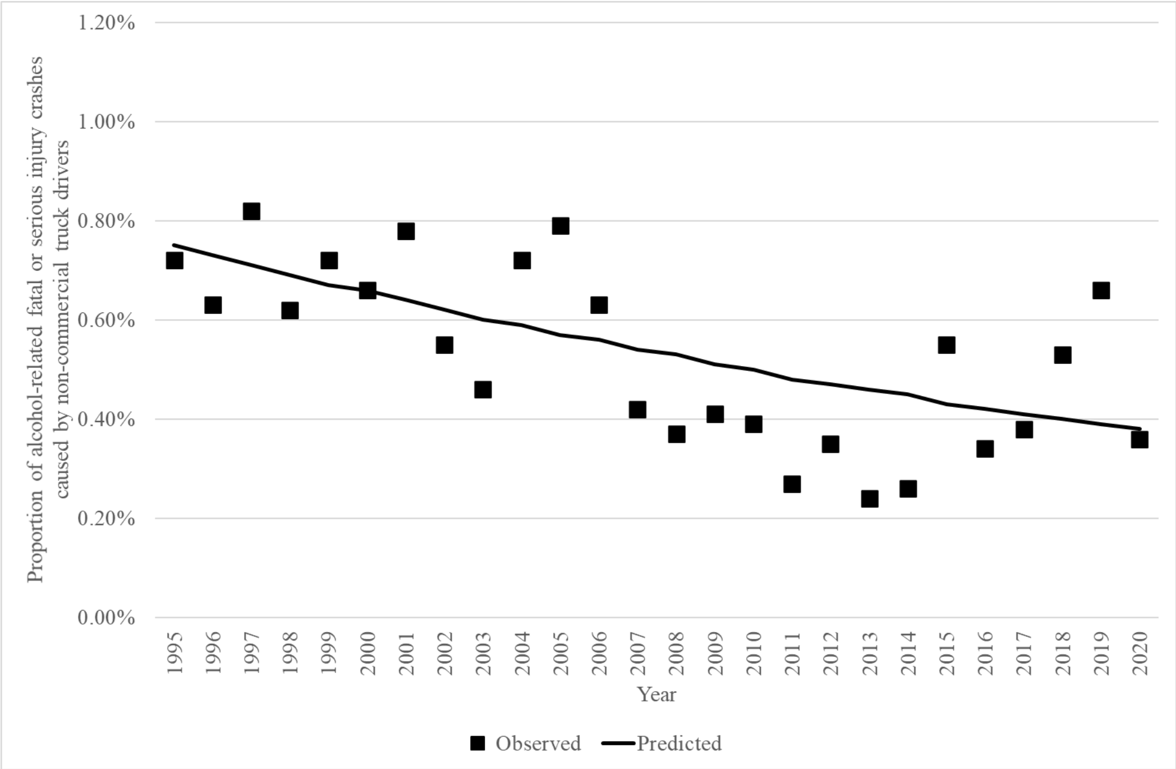

1.20%
1.00%
0.80%
0.60%
0.40%
0.20%
0.00%
Proportion of alcohol-related fatal or serious injury crashes caused by non-commercial truck drivers
1995
1996
1997
1998
1999
2000
2001
2002
2003
2004
2005
2006
2007
2008
2009
2010
2011
2012
2013
2014
2015
2016
2017
2018
2019
2020
Year
Observed
Predicted

(C)

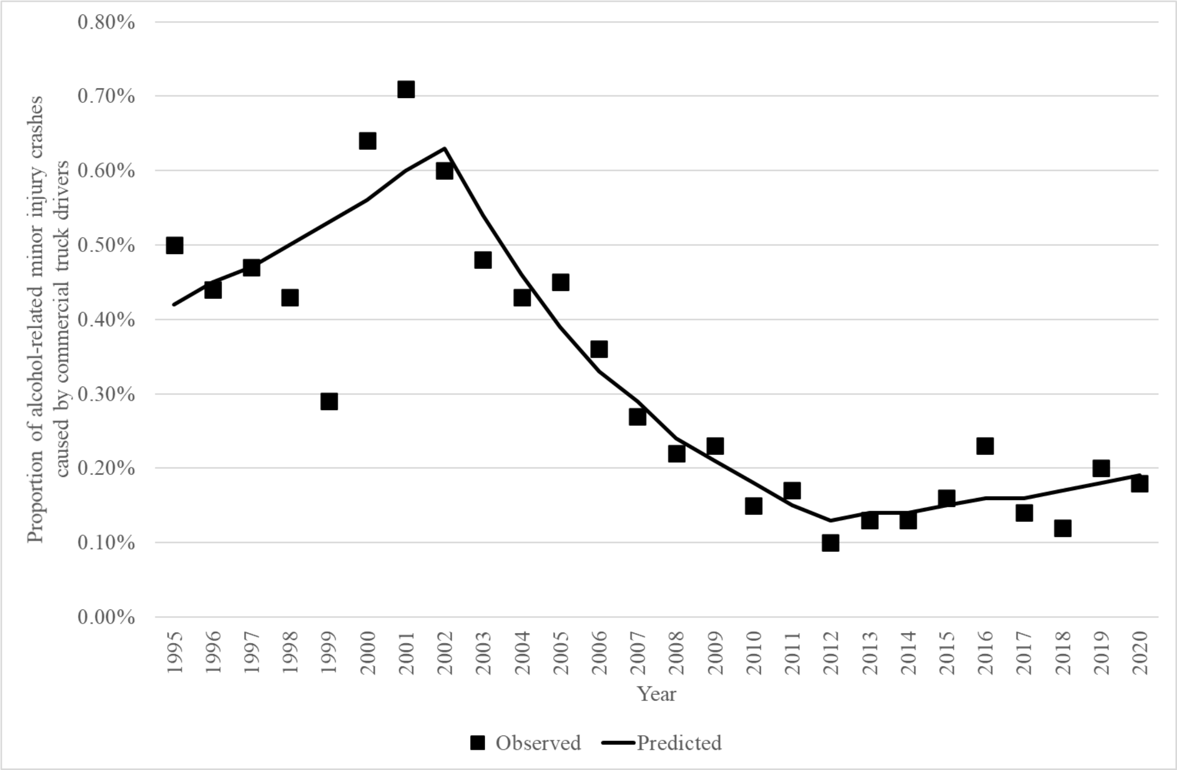

(D)

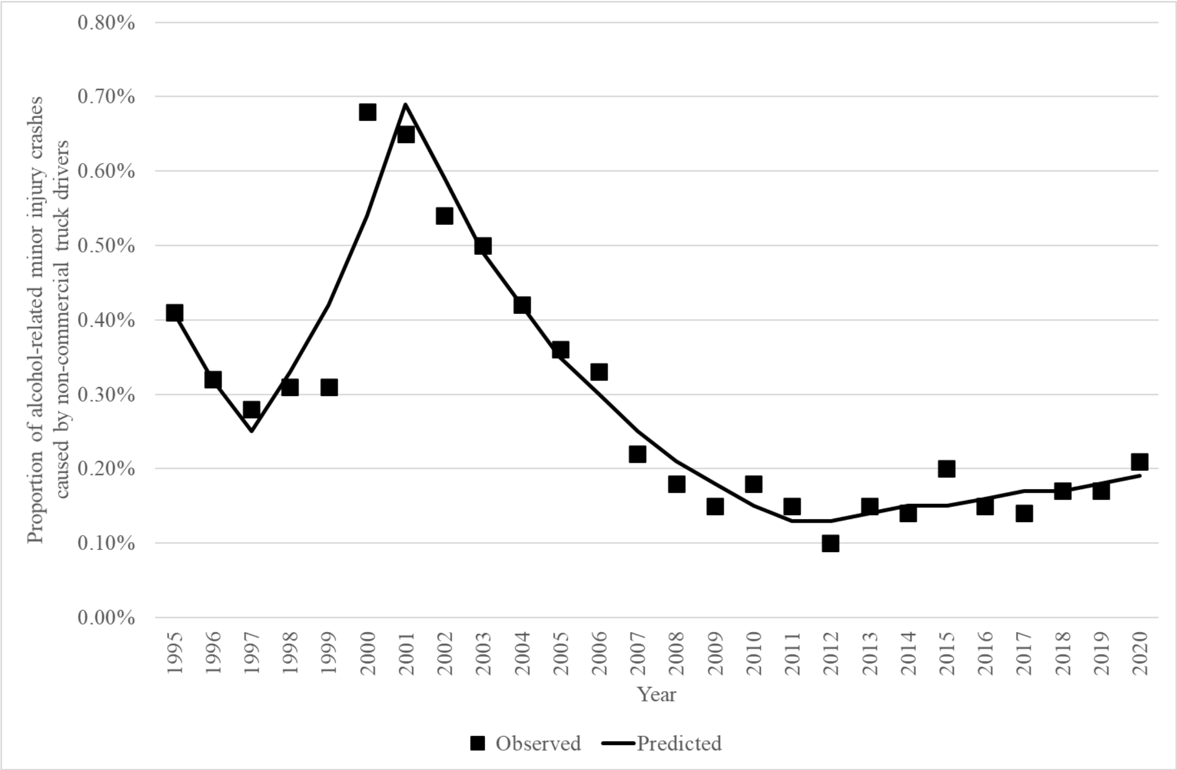

eFigure 2. Observed and predicted trend (dots and solid line) in the proportion of alcohol-related fatal or serious injury crashes caused by (**A**) commercial truck drivers and (**B**) non-commercial truck drivers, and that of alcohol-related minor injury crashes caused by (**C**) commercial truck drivers and (**D**) non-commercial truck drivers from 1995–2020. During the 2000s, several legal amendments were made against drunk driving. Since May 2011, commercial truck drivers have been required to take alcohol breath tests at the beginning and end of their working hours.

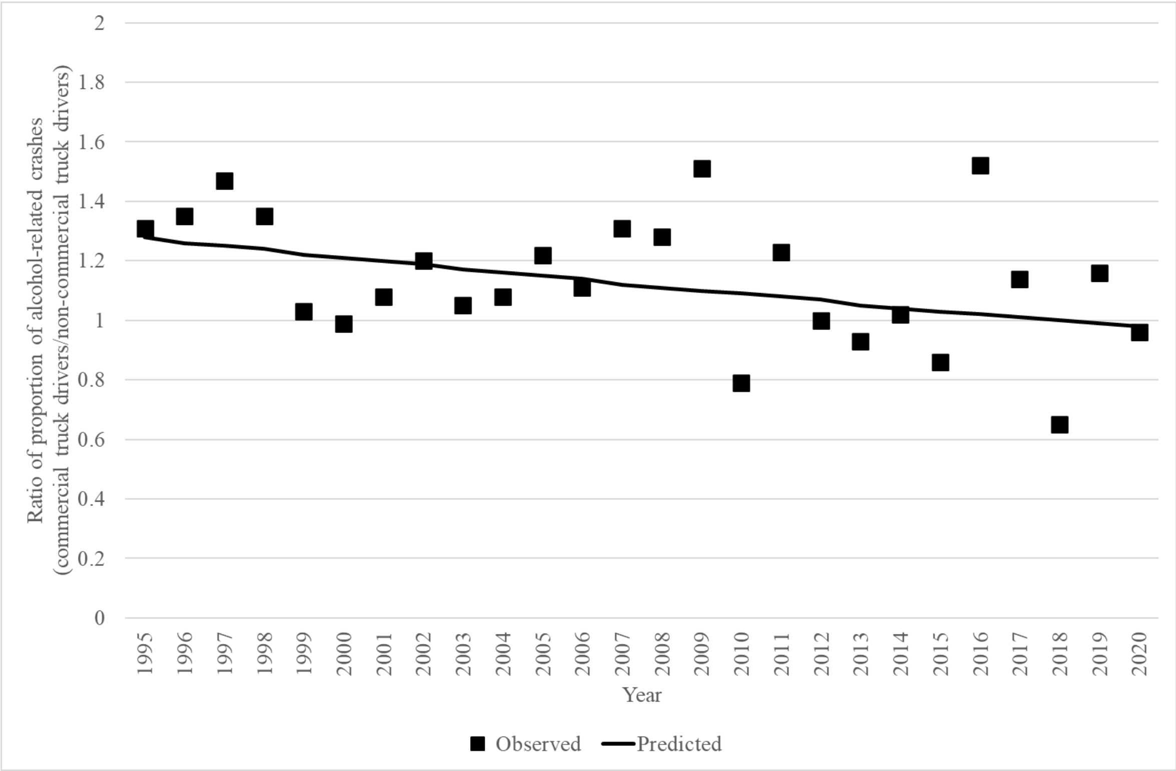

eFigure 3. Observed and predicted trend (dots and solid line) in the ratio of the proportion of alcohol-related crashes caused by commercial truck drivers to those caused by non-commercial truck drivers from 1995–2020. During the 2000s, several legal amendments were made against drunk driving. Since May 2011, commercial truck drivers have been required to take alcohol breath tests at the beginning and end of their working hours. All vehicle drivers include four-wheeled vehicles, motorcycles, and moped drivers.
